# Psychometric properties of the Perceived Stress Scale (PSS-10) in silica-exposed workers from diverse cultural and linguistic backgrounds

**DOI:** 10.1186/s12888-024-05613-6

**Published:** 2024-03-05

**Authors:** Fiona Hore-Lacy, StellaMay Gwini, Deborah C. Glass, Christina Dimitriadis, Javier Jimenez-Martin, Ryan F. Hoy, Malcolm R. Sim, Karen Walker-Bone, Jane Fisher

**Affiliations:** 1 Monash University, Melbourne, Australia; 2 Alfred Health, Melbourne, Australia

**Keywords:** Perceived stress, Silicosis, Occupational health, Psychometric properties, Culturally and linguistically diverse, Interpreters

## Abstract

**Background:**

The Perceived Stress Scale (PSS-10) has been used in a range of occupational cohorts, but only recently in stone benchtop workers undergoing screening for silicosis. The aim of this study was to compare psychometric properties of the PSS-10 in stone benchtop workers amongst those born overseas or who used an interpreter.

**Methods:**

Stone benchtop workers in Melbourne, Australia completed the PSS-10 as part of their occupational screening for silicosis. Internal consistency was assessed with Cronbach’s α for the total score and the positive and negative subscales. Validity was assessed using confirmatory factor analysis (CFA). Analysis was performed for the total group and for subgroups according to sex, interpreter use, overseas-born, and language spoken at home.

**Results:**

The results of 682 workers with complete PSS-10 scores were included in analysis. Most participants were male (93%), with mean age 36.9 years (SD 11.4), with just over half (51.6%) born in Australia, 10.1% using an interpreter, and 17.5% using a language other than English at home. Cronbach’s α for the overall group (α = 0.878) suggested good internal consistency.

**Discussion:**

CFA analysis for validity testing suggested PSS-10 performance was good for both sexes, moderate for country of birth and language spoken at home categories, but poorer for those who used an interpreter. Whilst professional interpreters provide a range of benefits in the clinical setting, the use of translated and validated instruments are important, particularly in cohorts with large numbers of migrant workers.

**Conclusion:**

This study describes the psychometric properties of the PSS-10 in a population of stone benchtop workers, with good internal consistency, and mixed performance from validity testing across various subgroups.

## Introduction

The aim of this study was to compare the psychometric properties of the Perceived Stress Scale (PSS-10) amongst workers in the stone benchtop industry attending for screening, specifically exploring the properties amongst people who spoke a language other than English at home or who used an interpreter.

Stress is a psychological phenomenon associated with a behavioural and physiological response to an opportunity, challenge or threat. Whilst acute stress is part of basic survival, chronic stress can cause significant harms to health [1, 2]. Stress perception is defined as the psychological response to stress [3]. One of the most widely-used measures of stress perception is the PSS [4], which instead of focussing on a specific event or stressor, measures the stress a person attributes to their life [5]. It was designed as a way to quantitate past, present, and future, or anticipated, stress, and the tool encompassed its unpredictable, uncontrollable, and overloading nature. The original PSS included 14 items (PSS-14) but has since been refined into a 10-item version (PSS-10) which has been translated and validated extensively for use in many countries, including in: Europe (France [6], Spain [7], Germany [8], Serbia [9]; South America (Brazil [10], Mexico [11]); USA [12]; China [13]; and South East Asia (Korea [14], Vietnam [15] Thailand [16], and Bangladesh [17]).

Silicosis is an irreversible, fibrotic lung disease which arises following inhaled exposure to respirable crystalline silica (RCS). Crystalline silica is found in sand and most types of rock and is therefore ubiquitous in the earth. Traditionally, significant RCS exposure was most common among workers within industrial sectors like mining and construction. However, an epidemic of silicosis has emerged amongst workers cutting or grinding artificial stone [18, 19]. Artificial stone is manufactured worldwide, and has silica content of up to 90%, far higher than that of natural stones (marble 3%, granite 30% on average) [20]. Internationally, cases of silicosis in the stone benchtop industry have been reported since 2010. Artificial stone arrived in Australia in the early 2000s and the first cases were reported in 2015 [19, 21, 22]. In response to the increasing number of patients presenting to the health system with silicosis, WorkSafe Victoria, the state occupational safety and health regulator, established a free health screening service for workers in the stone benchtop industry. Among the tools administered during screening was the PSS-10 instrument.

Psychometric properties of the PSS-10 have been reported in a range of occupational settings such as public sector, healthcare, and education, as shown in Table 1, but not among artificial stone benchtop workers.

**Table 1:** Psychometric properties of the PSS-10 in occupational settings

Reference and year	Occupation of participants (Language of instrument)	Number in study^a^ (%male)	Age in years (mean (SD))	Factorial validity			Cronbach ɒ		
				Factors	Total %	(Negative % + Positive %)	Overall	Negative	Positive
Reis et al. 2010 [10]	University teachers in Brazil (Portuguese)	793 (57.5%)	45.5 (8.0)	2	56.8%	30.6% + 26.1%	0.87	0.83	0.77
Wang et al. 2011^b^ [23]	Policewomen in China (Chinese)	240 (0%)	21.1 (1.4)	2	62.41%	47.61% + 14.80%	0.86	0.87	0.77
Lesage et al. 2012^c^ [6]	Workers in occupational health care centres in France (French)	501 (49.7%)	40.4 (UNK)	2	55%	-	0.83	0.81	0.73
Ng et al. 2013 [24]	Elderly service workers in Hong Kong (Chinese)	992 (16.5%)	43.2 (10.2)	2	49.1%	29.15% + 19.95%	0.70	0.78	0.67
Smith et al. 2014 [25]	Accountants in USA (English)	305 (49.7%)	32.9 (12.5)	-	-	-	0.857	0.847	0.842
Sandhu et al. 2015 [26]	Nurses in Malaysia (Malay)	229 (UNK%)	48.3 (5.6)	2	54.6%	32.1% + 22.5%	0.63	0.82	0.72
Miranda et al. 2020 [27]	Public sector workers in Argentina (Spanish)	535 (20.4%)	Categorical age reported	2	60%	33.5% + 26.5%	0.810	0.832	0.806
Teresi et al 2020^d^ [28]	Hispanic caregivers to patients with Alzheimer’s in USA (Spanish, English)	453 (16%)	58.4 (11.2)	2	64.8%	53.7% + 11.1%	0.88	-	-

^a^Where both EFA and CFA was performed, total population was included in this column

^b^No mention of reverse-scoring positive items in methodology

^c^Reverse-scored items are incorrectly described as ‘negative’ items instead of ‘positive’, however the item numbers described as reverse-scored correlate to positive items

^d^Authors conclude data fits unidimensional model due to ratio of two factors. Eigenvalues reported in Supplementary Material

In an earlier study amongst this cohort, we found that higher perceived stress was associated with dyspnoea; having left the industry; attending clinic early in the screening period; being referred to a respiratory specialist; and a history of anxiety or depression [29]. However, we found that interpreter use and being born overseas were associated with lower perceived stress scores [29]. Whilst it was possible that these workers did indeed have lower levels of perceived stress than other workers, another hypothesis was that the psychometric properties of the tool itself were affected by language and/or interpreter use. Of course, the use of interpreters in the clinical setting is a valuable element to optimise patient care for people from diverse backgrounds [30]. However, there is some evidence that when completing validated questionnaires, interpreter use may impact the scores obtained. For example, in a study using the Edinburgh Postnatal Depression Scale, it was observed that comprehension of interpreted questions might be limited if they contained “Western” terminology or concepts, which resulted in rewording of questions to culturally-appropriate concepts [31].

## Methods

The study used cross-sectional data from baseline questionnaires to establish the psychometric properties of PSS-10.

### Participants

People from the stone benchtop industry were offered optional free screening for silica-associated disease. Details of the assessment, including use of the Perceived Stress Scale, has been previously described [29, 32, 33]. Assessments were conducted between July 2019 and September 2021, with written consent to share data with Monash University obtained during the screening. Only workers with complete PSS-10 data were included in these analyses.

### Perceived stress scale

The PSS-10 is a 10-item questionnaire with each item scored on a 5-point Likert scale (0 = never, 4 = very often). A high score indicates a high level of perceived stress, with a maximum score of 40. Six of the questions are worded negatively (e.g. How often have you been upset because of something that happened unexpectedly?) and are scored as is. The other 4 questions are worded positively (e.g., How often have you been able to control irritations in your life?), and are reverse-scored (i.e. rating of 0 scored as 4).

Subscales can be calculated [34], with negatively-worded items used for a negative subscale that has a maximum score of 24, with a high score indicating a high level of distress. The reverse-scored positively-worded items can be used for a positive subscale, that has a maximum score of 16, with a high score indicating a high level of coping.

### Variables of interest

Language used at home, country of birth, and interpreter use were recorded by the occupational physician during the assessment.

### Data analysis

Internal consistency (to assess how inter-related items within a scale are) was measured using the Cronbach’s ɒ coefficient, using Stata Statistical Package v17 [35]. A Cronbach’s α ≥ 0.9 is usually defined as “excellent” consistency; 0.8–0.9, “good” and 0.7–0.8 “acceptable” [36].

Validity based on the internal structure of the PSS-10 was assessed using confirmatory factor analysis (CFA) using Mplus v8.10 [37]. Previous psychometric testing [5], including that summarised in Table 1, suggested that the PSS-10 comprised two factors: one constituting the positively-worded questions (Positive factor) and the other constituting the negatively-worded questions (Negative factor). Therefore, this model was tested in the current CFA. The model parameters assessed in the CFAs included the following goodness‐of‐fit indices: chi-squared test; comparative fit index (CFI), the root mean square error of approximation (RMSEA), the standardised root mean square residual (SRMR) and the Tucker-Lewis Index (TLI). Values of the CFI above or close to 0.95, SRMR values below 0.08, and RMSEA values below 0.06 were interpreted as a good fit [38]. RMSEA values between 0.06 and 0.08 were interpreted as a reasonable fit [39].

Factor invariance (comparison of factor model fitness across subgroups) was assessed by first estimating the configural invariance (comparison of the factor construct) Chi-square statistic, followed by calculating the change in Chi-squared statistic (and *p*-value for hypothesis H_0_: change in χ^2^ = 0) for metric invariance (i.e. test of whether the subgroups interpret the questions in a similar way) and scalar invariance (i.e. test whether the subgroups have the same means on the latent variables for each factor). Model invariance testing was only conducted if model fit for each of the subgroups was acceptable. The model was concluded as invariant based on the model fit parameters, RMSEA, SRMR, TLI and CFI using the rules described above.

Both Cronbach’s α and CFA analyses were completed for the overall group, as well as subgroups based on country of birth and interpreter use. For all analyses, statistical significance was determined using α = 0.05.

Ethics approval for the study was granted by Monash University Human Research Ethics Committee (reference 17730).

## Results

In total, 717 silica-exposed workers attended for health screening and completed a PSS-10, amongst whom 682 (98.6%) provided complete PSS responses. The participants were mostly (93%) male, mean age 36.9 years (SD 11.4). Just over half (51.6%) were born in Australia and 69 (10.1%) used an interpreter when completing their PSS-10 as reported in Table 4. PSS-10 scores and subscale scores are reported in Table 2.

**Table 2:** Worker demographics

	N (%)
All participants	682 (100%)
**Sex**	
Male	633 (92.8%)
Female	49 (7.2%)
**Country of birth**	
Australia	352 (51.6%)
Overseas	307 (45.0%)
Not stated	23 (3.4%)
**Interpreter used for consultation**	
Interpreter	69 (10.1%)
No interpreter	522 (76.5%)
Not stated	91 (13.3%)
**Language spoken at home**	
English	486 (71.3%)
Other	119 (17.5%)
Not stated	77 (11.3%)

### Internal consistency

Internal consistency indicated that the items within the PSS-10 were closely related for all participants, with a Cronbach’s α of 0.878 (Table 3). The consistency for the two subscales was also good with Cronbach’s α of 0.882 and 0.751 for the negative and positive subscales respectively. The internal consistency remained high when analysed in subgroups: by gender; country of birth; interpreter use and language spoken at home (Table 3).

**Table 3:** PSS-10 subscales

	N	Total score (Mean ± SD)	Negative subscale (Mean ± SD)	Positive subscale (Mean ± SD)
All participants	682	12.7 ± 7.0	7.5 ± 5.0	5.2 ± 3.1
Male	633	12.7 ± 7.0	7.5 ± 5.1	5.3 ± 3.2
Female	49	12.3 ± 6.4	8.0 ± 4.8	4.3 ± 2.5

PSS-10 maximum score: 40 (high score indicates a high level of perceived stress). Negative subscale maximum score: 24 (high score indicating a high level of distress). Positive subscale maximum score: 16 (high score indicating a high level of coping)

### Validity

The results of the validity testing by CFA suggested that the PSS-10 performed well in both sexes, moderately well with country of birth Australia vs. non-Australia and language spoken at home categories (English vs. other), but less well among those who used an interpreter during the consultation (Table 4). The results of the CFI, TLI and SRMR showed an acceptable model fit for both males and females, those born in Australia or overseas, those who did not use an interpreter, and those who speak English at home. However, an acceptable model fit could not be established for participants who used an interpreter. In all subgroups, the RMSEA suggested only a mediocre level of fit.

**Table 4:** Psychometric properties of PSS-10 amongst stone benchtop workers in whole sample and by gender, country of birth, interpreter use and language spoken at home

	N (%)	CONSISTENCY (Cronbach’s alpha)						VALIDITY (Confirmatory factor analyses)			
		PSS-10 items	Subscales		Goodness-of-fit			RMSEA (90% CI)	CFI	TLI	SRMR
			-ve	+ve	χ^2^	χ^2^/df	Δχ2 (Δdf) *p*-value				
**All participants**											
	682 (100%)	0.878	0.882	0.751	142.42	4.19	-	0.068 (0.057–0.080)	0.988	0.984	0.022
**By sex**											
Male	633 (92.8%)	0.880	0.883	0.751	154.87	4.56	-	0.075 (0.063–0.087)	0.986	0.982	0.023
Female	49 (7.2%)	0.859	0.856	0.729	34.44	1.01	-	0.016 (0.00-0.105)	0.999	0.999	0.059
Configural invariance	-	-	-	-	145.09	2.13	-	0.058 (0.045–0.071)	0.992	0.989	0.028
Metric Invariance	-	-	-	-	151.85	2.00	12.886 (8) *p* = 0.116	0.054 (0.041–0.067)	0.991	0.991	0.029
Scalar invariance	-	-	-	-	157.19	1.56	32.160 (33) *p* = 0.509	0.040 (0.028–0.052)	0.994	0.995	0.031
**By country of birth**											
Australia	352 (51.6%)	0.895	0.879	0.787	77.07	2.27	-	0.060 (0.42 − 0.078)	0.992	0.989	0.025
Overseas	307 (45.0%)	0.860	0.877	0.720	96.49	2.84	-	0.077 (0.059–0.096)	0.983	0.977	0.030
Not stated	23 (3.4%)	-	-	-	-	-	-	-	-	-	-
Configural invariance	-	-	-	-	179.84	2.64	-	0.071 (0.058–0.083)	0.988	0.984	0.027
Metric Invariance	-	-	-	-	195.85	2.58	22.469 (8) *p* = 0.004	0.069 (0.057–0.081)	0.987	0.984	0.029
Scalar invariance	-	-	-	-	243.90	2.35	81.428 (36) *p* < 0.001	0.064 (0.054–0.074)	0.985	0.987	0.033
**By interpreter use**											
Interpreter	69 (10.1%)	0.824	0.851	0.658	88.05	2.59	-	0.152 (0.113–0.191)	0.940	0.921	0.070
No interpreter	522 (76.5%)	0.884	0.883	0.770	83.91	2.47	-	0.053 (0.039–0.067)	0.993	0.991	0.020
Not stated	91 (13.3%)	-	-	-	-	-	-	-	-	-	-
**By language spoken at home**											
English	486 (71.3%)	0.882	0.884	0.737	82.81	2.44	-	0.054 (0.40-0.069)	0.993	0.991	0.020
Other	119 (17.5%)	0.869	0.874	0.762	102.03	3.00	-	0.130 (0.101–0.159)	0.948	0.931	0.054
Not stated	77 (11.3%)	-	-	-	-	-	-	-	-	-	-

Since the model fit for the two sex and country of birth subgroups were acceptable, model invariance was tested for these two variables. For both variables, the model fit for configural, metric and scalar invariance satisfied at least two of the model goodness-of-fit parameters and the change in Chi-square statistic was small (*p* > 0.05), therefore indicating complete model invariance for sex and country of birth. Model invariance was not estimated for language spoken at home and interpreter use because the model for at least one of the subgroups did not achieve good model fit.

## Discussion

This study assessed the psychometric properties of the PSS-10 in a novel occupational cohort of stone benchtop industry workers, in order to understand how the instrument performed across different demographics of participants. The consistency of the measure was generally good for different subsets of the population, including gender and English as first language vs. not. However, assessment of validity indicated that instrument validity differed by language spoken at home and use of interpreter, with poorer performance among those who spoke a language other than English at home and those who used an interpreter. Compared to the studies referenced in Table 1, the psychometric properties were found to be similar amongst this specific cohort, including a lower score in the positive subscale compared to the negative subscale.

Our findings need to be considered alongside some limitations. Firstly, the current study did not include any questions about educational attainment. It has been found in other studies that people with poorer educational attainment report higher stress scores on the PSS-10 [40]. A Spanish qualitative study of ten artificial stone benchtop workers diagnosed with silicosis found that five had elementary school education and four had middle school attainment, with only one with tertiary education [41]. Given that educational data was not collected, it is impossible to know if the current results could be explaiend by differences in education.

Secondly, because this was analysis of “real world” clinical data, only one mental health instrument was utilised at a single time point. Therefore, validity testing using other mental health instruments or test-retest reliability analysis using repeat measures were not able to be performed. Thirdly, as the screening was not mandatory, those included in this analysis did not represent all workers in Victoria. It is possible that migrant workers are under-represented in this analysis, which also may limit the generalisability of the results. Finally, the sample included few women, which may limit the generalisability of the results for female workers.

The advantages of this study were that we included a large sample of workers from the stone benchtop industry and had complete data available for over 98% clinic attendees, minimising the chances of any responder bias. Given the nature of the work and workers involved, our results contribute new understanding about the use of a measure like PSS-10 in “real world” settings.

As a result of globalisation, migrant populations have increased in many countries, so that a growing proportion of the population of countries like UK, USA and Australia do not speak English at home. In Australia, the 2016 Census revealed that 21% of Australians spoke languages other than English at home [42], which is reflected in our data. Systematic reviews demonstrate that the resultant language barriers affect physician and patient satisfaction, quality of care, complications from care, patient safety, delay in diagnosis and treatment affecting length of stay and increased costs of healthcare [30, 43, 44]. Increasing the provision of professional interpreters for healthcare settings might mitigate these negative effects by decreasing the use of informal interpreters such as friends or family members who are not professionally-bound to confidentiality policies, and may have difficulty understanding or interpreting complex medical terminology. The benefits of using a professional interpreter in health settings has been well-established [30, 45]. Less is known about the use of interpreters for a measure like PSS-10, which may be influenced by the language and culture of both the interpreter and the client.

A study using the International Trauma Questionnaire (ITQ) to measure post-traumatic stress disorder (PTSD) amongst refugees reported on the psychometric and validity measures of its use in three languages (Danish, Arabic, Bosnian) and a with an interpreter [46]. The study also accounted for degrees of assistance required by responders. It measured the differential item functioning (DIF) for the categories of age, gender, ITQ language (or interpreter), assistance from interpreter or clinician, and years from traumatic exposure. No DIF was identified for ITQ language or interpreter use, indicating that the psychometric properties for this category were stable. Interestingly, interpreter assistance was also recommended where needed, as long as the interpreters had been trained in administering the ITQ. Assessing the psychometric properties of the PSS-10 when administered with interpreters who are trained for the instrument could be a topic for future research.

Another study involving a different PTSD instrument, the Child and Adolescent Trauma Screen (CATS), assessed the prevalence of post-traumatic stress disorder amongst refugees to Germany, and reported on psychometric properties amongst those who used an interpreter to complete the questionnaire compared to those who used a validated translation of the questionnaire [47]. Although Cronbach α scores for the overall CATS score did not differ significantly between the two groups, the authors cautioned against using interpreters due to the challenges of a clinical setting compared to the ‘elaborate’ and ‘iterative’ process of a validated translation. A validated translation requires the questionnaire wording to be translated and back-translated, with the psychometric properties analysed and compared to the original. It may then require its scores to be interpreted specifically for its context [48]. In contrast, an interpreter performs a ‘live translation’ in clinic, closely following instructions, including those regarding paraphrasing and repetition [49]. It is important to be able to standardise the administration of an instrument if an interpreter is involved, in order to be able to compare the psychometric properties to other settings or languages [31]. We previously reported lower PSS-10 scores amongst those born overseas [29], and now this psychometric analysis appears to suggest lower validity in those who used an interpreter or spoke a language other than English at home. When instrument scores, like the PSS-10, affect clinical decisions, it is particularly important to have appropriate instruments.

Migrant workers are recognised to be at higher risk of accidents and injuries and hazardous workplace exposures than non-migrant workers [50, 51]. Moreover, they are less likely to raise concerns about workplace safety, and more likely to work without sufficient training or protection for longer hours and less pay [50, 52]. Where workplace saftey training is provided, it may not be in a language spoken by the worker [51]. Their vulnerable status was summarised by one author as “dirty, dangerous, and demanding (sometimes degrading or demeaning)” [50]. One Australian study found that Vietnamese workers were more likely to report jobs with high demands and low control than Australian-born workers, and that Australian-born workers were less likely than migrant workers to be in low-security jobs [53]. The vulnerable status of migrant workers underlines the need to accurately measure psychosocial factors.

In summary, we evaluated the psychometric properties and internal consistency of the PSS-10 amongst an occupational cohort including a high proportion of migrant workers and workers amongst whom English was not the first language. Generally, the PSS-10 showed reasonable internal consistency and good validity but its performance with an interpreter and amongst non-English speakers was weaker. In the future, tools that have been validated in the population under study, or for which translated tools have been validated, are recommended. Further research could usefully clarify whether the poorer performance of PSS-10 related to an interpreter effect or the translation itself.

## Data Availability

Patient consent did not include sharing of data external to Monash University. If data needs to be shared, additional approval from Monash University HREC would be required. The datasets generated and analysed during the current study are not publicly available due to ethical and privacy reasons, but are available from the corresponding author on reasonable request.
